# The Royal Portsmouth Hospital

**Published:** 1912-03-16

**Authors:** T. H. F. Lapthorn

**Affiliations:** Chairman of the Committee of Management. Royal Portsmouth Hospital, Portsmouth


### The Royal Portsmouth Hospital.

*To the Editor of* The Hospital.

Sir,—In your last issue you start a very adverse criticism of the administration of this hospital with the words, "For several years the financial and general condition of the Royal Portsmouth Hospital has been going downhill," and say that our resources have been diminishing since 1907. In fairness to those responsible for the administration, I ask you to insert the following reply.

First, as to the financial position. In the five years 1907-11, we have opened a new operating theatre, corridors, two children's wards, and a nurses' home, and installed an electric lift, at a total cost of £19,850. The whole of this has been raised and paid for by voluntary gifts, without touching our invested funds, except to the extent of £3,845, and this sum we hope to replace during this year. We already have a promise of £2,000 towards it, conditional upon the balance being raised by the end of the year. In addition, we have during the same period opened a new out-patients' department, the cost of which, amounting to £4,583, was borne by one of our governors.

It is true, as you say, that our annual subscriptions last year were £82 below 1907, but our ordinary income last year, from all sources, was slightly in excess of 1907, and was, in fact, the highest that we have ever had in any year, with one exception. And the reason why our income has not been more elastic during the last few years is, I submit, quite obvious from the figures which I have given above as to the expenditure on rebuilding and extensions.

You also assume, contrary to the fact, that we have grave differences of opinion among us, and suggest that "the cause of the trouble" lies in "an unwise and narrow policy in the management of its affairs." I prefer that the reply to this suggestion of a lack of confidence in the management, on the part of the subscribers, should be made by one who is no way connected with the management, but has always shown himself a keen, though friendly, critic of it. I therefore enclose an extract from the *Portsmouth Evening News* of the 8th inst.

By far the greater part of your article of two columns is an attack upon the governors, because a resolution was proposed—not carried—to allow the members of the senior honorary staff to dispense; and it is not until you get to the bottom of the second column that you mention the important fact that the resolution was defeated. It was, in fact, defeated by a considerable majority, but surely it is not fair criticism to denounce the management of a hospital because a resolution of which you strongly disapprove was brought forward, when you knew, when writing the criticism, that the resolution was not carried. —Yours faithfully,

T. H. F. Lapthorn.

Chairman of the Committee of Management.

Royal Portsmouth Hospital, Portsmouth,
March 9.

[*** We are glad to publish Mr. Lapthorn's letter and to find that the extraordinary expenditure during 1907-11 has been covered by extraordinary income with the exception of £3,845. This is most satisfactory. We dealt, however, with the ordinary income, and especially with the receipts from annual subscriptions and Hospital Sunday, and a deficiency of £467 for the year 1911. Mr. Lapthorn does not question the accuracy of the figures we quoted from the annual report published in the *Portsmouth Times* of the 2nd instant. Our point was that the ordinary income of this hospital is much too small, having regard to the population of Portsmouth and Southsea, if the hospital had behind it the united goodwill and support of all classes of residents. The extract from the *Portsmouth Evening News* referred to by the chairman states : "We doubt if there ever was a time in the history of the hospital when the management policy commanded more fully the confidence of the public. . . The improved system of management was revolutionised a few years ago, and the present system is thoroughly democratic." The *Portsmouth Evening News* adds : "The Southsea residents have had an opportunity of taking part in the management which has been knocking at their doors for years." The chairman has missed our point, which was that at a meeting on January 5 last the governors by a large majority had carried the resolution referred to in the last paragraph of his letter, but this decision was not confirmed at the meeting of the 2nd instant, owing to the able speech and efforts of Mr. Rundle, the consulting surgeon. We are glad to have the chairman's assurance that there are no grave differences of opinion at the present time, and whilst thanking him for his letter, we venture to hope that one result of this discussion will be that the ordinary income from all sources during 1912 will be much increased and so worthier of the large population the needs of which this hospital ministers.—Ed. The Hospital.]

